# Graphene–carbon 2D heterostructures with hierarchically-porous P,N-doped layered architecture for capacitive deionization†

**DOI:** 10.1039/d1sc00915j

**Published:** 2021-06-30

**Authors:** Jingru Guo, Xingtao Xu, Jonathan P. Hill, Liping Wang, Jingjing Dang, Yunqing Kang, Yuliang Li, Weisheng Guan, Yusuke Yamauchi

**Affiliations:** School of Water and Environment, Chang'an University, Key Laboratory of Subsurface Hydrology and Ecological Effects in Arid Region, Ministry of Education Xi'an 710064 P. R. China guanweisheng123@163.com; JST-ERATO Yamauchi Materials Space-Tectonics Project, International Center for Materials Nanoarchitectonics (WPI-MANA), National Institute for Materials Science (NIMS) 1-1 Namiki Tsukuba Ibaraki 305-0044 Japan XU.Xingtao@nims.go.jp; College of Geology and Environment, Xi'an University of Science and Technology Xi'an 710054 PR China; Australian Institute for Bioengineering and Nanotechnology (AIBN), The University of Queensland Brisbane QLD 4072 Australia y.yamauchi@uq.edu.au

## Abstract

Exploring a new-family of carbon-based desalinators to optimize their performances beyond the current commercial benchmark is of significance for the development of practically useful capacitive deionization (CDI) materials. Here, we have fabricated a hierarchically porous N,P-doped carbon–graphene 2D heterostructure (denoted NPC/rGO) by using metal–organic framework (MOF)-nanoparticle-driven assembly on graphene oxide (GO) nanosheets followed by stepwise pyrolysis and phosphorization procedures. The resulting NPC/rGO-based CDI desalinator exhibits ultrahigh deionization performance with a salt adsorption capacity of 39.34 mg g^−1^ in a 1000 mg L^−1^ NaCl solution at 1.2 V over 30 min with good cycling stability over 50 cycles. The excellent performance is attributed to the high specific surface area, high conductivity, favorable meso-/microporous structure together with nitrogen and phosphorus heteroatom co-doping, all of which are beneficial for the accommodation of ions and charge transport during the CDI process. More importantly, NPC/rGO exhibits a state-of-the-art CDI performance compared to the commercial benchmark and most of the previously reported carbon materials, highlighting the significance of the MOF nanoparticle-driven assembly strategy and graphene–carbon 2D heterostructures for CDI applications.

## Abstract 2

MOF nanoparticle-driven assembly on 2D nanosheets produces the graphene–carbon heterostructure with hierarchically-porous P,N-doped layered architecture.

## Graphical abstract

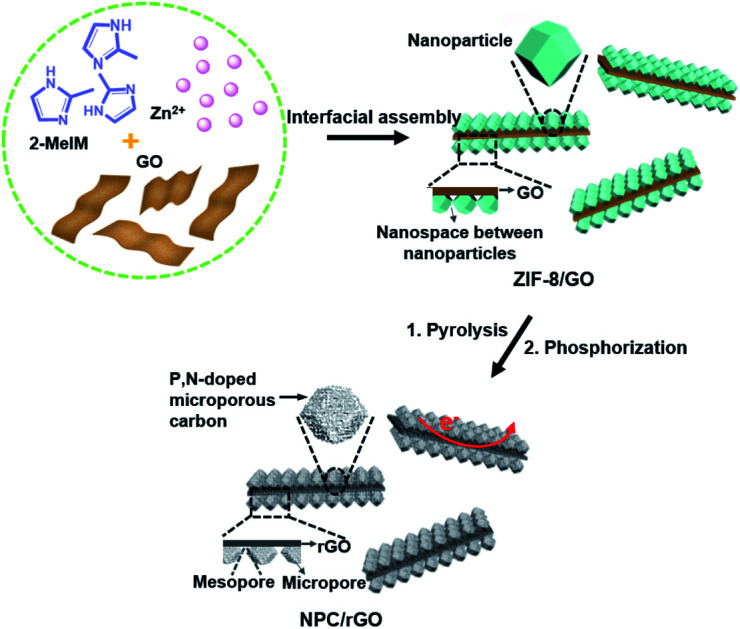

## Introduction

With an increasing world population, worsening environment, and climate and energy crises,^1–4^ the economical supply of clean, potable water is rapidly becoming a critical issue for human subsistence. In this regard, capacitive deionization (CDI), an advanced electrochemical deionization technology, has emerged as a competitive methodology for the desalination of brackish water and seawater, due to its several important advantages including low energy consumption, cost effectiveness, and environmental benignity.^5–9^ The working principle of CDI is similar to that of electric double layer (EDL) capacitors,^10^ where the application of a low directional current causes charged ions to approach the oppositely charged electrode, with their subsequent adsorption at internal pores by formation of EDLs. This process eventually provides purified water with extracted ions being continuously stored within the electrode materials. An ideal CDI electrode possesses large specific surface area (SSA), rational pore structure, excellent electrical conductivity and wettability, and good electrochemical stability.^11–13^ Accordingly, there has been substantial effort focused on the synthesis of porous carbon materials having appropriate characteristics to achieve optimal CDI performance.^14–18^ Unfortunately, however, most porous carbon materials obtained by simple processing routes exhibit poor salt adsorption capacity (SAC) because of their limited functionality, which leads to a sparsity of charged groups at their surfaces, narrow pore size distribution and low electrical conductivity,^19^ although they can have very large SSAs and highly microporous structures. The application-oriented design of nanostructured porous carbon is therefore critical for the practical scale-up CDI applications.

Recent work has demonstrated that the incorporation of heteroatoms such as nitrogen (N), sulphur (S) and phosphorus (P) into a carbon matrix is an effective strategy to augment the surface functionalities of carbon materials.^20–22^ This would not only improve the wettability and electron transfer efficiency, but also introduce unique metal-cation-coordinating properties for enhanced CDI performance.^23^ Li *et al.* prepared electrodes based on an N-doped hierarchical porous carbon exhibiting SAC of 13.76 mg g^−1^ in 500 mg L^−1^ NaCl solution, representing more than 30% improvement over the non-doped carbon material.^24^ Pan *et al.* obtained a high SAC of 16.20 mg g^−1^ at 1.2 V in 1000 mg L^−1^ NaCl solution using P-doped 3D carbon nanofiber aerogels as CDI electrodes, also significantly improving on the undoped carbon materials (12.81 mg g^−1^).^25^ Hu *et al.* reported an asymmetric CDI cell using N-doped activated carbon (AC) and AC that has a maximum reversible SAC of around 24.7 mg g^−1^ and improved cycling stability.^26^ Furthermore, multi-heteroatom-doped carbon materials ought to have higher values for SAC than carbons doped with single types of heteroatom, due to both the individual properties contributed by each doped heteroatom and to any synergistic effects occurring between the heteroatom dopants present.^27–29^ Despite the recent progress in multi-heteroatom doping methodology, the performances of the resulting carbon nanoarchitectures remains limited by their poor characteristics such as low SSA, which limits ion accommodation, unsuitable pore structure, which obstructs mass transport, and relatively low electrical conductivity, which negatively affects electron transfer, *etc.*^30^ Therefore, further modifications of the relevant carbon materials are required to address these challenging issues.

Carbon–carbon heterostructures are materials composed of two or more types of allotropic carbon species, which combines the advantages of each component but which might also possess superior properties not exhibited by the component species.^31^ Of the reported examples, graphene–carbon two-dimensional (2D) heterostructures containing a nanostructured carbon layer anchored at graphene nanosheets, have been widely studied because of their excellent electrical conductivity and unique 2D nanosheet structure.^32^ By careful tailoring of the morphology, structure and composition of the precursor coating layer, the functionalities of the resulting graphene–carbon 2D heterostructures could be tuned (*e.g.*, composition, pore structure).^33–35^ Furthermore, mesopore nanoarchitectonics of nanomaterials,^36^ especially in two dimensions, which is a set of methods aimed at engineering the mesopore architectures of 2D nanomaterials, has recently emerged as a hot research topic. The engineered mesopores in those materials might provide ion diffusion pathways and effectively enlarge their accessible surface areas for greater electrolyte access.^17^ The resulting highly mesoporous materials exhibit significantly improved electrochemical performances. Several strategies exist for this purpose including that reported by Kim *et al.* where a one-step, large-scale, low-cost, synthetic procedure involving *in situ* pyrolysis of mixed glucose, dicyandiamide and phosphoric acid was developed.^37^ A complicated, high-cost micelle-mediated methodology has also been widely studied for the engineering of mesopore architectures on graphene.^34^ Unfortunately, the 2D carbon materials resulting from that procedure lack sufficient micropores and have low porosity, which leads to poor performance especially in ionic adsorption applications. In this context, an improved understanding and the elaboration of strategies for the design and synthesis of 2D graphene–carbon heterostructures containing hierarchical meso–micropores is a significant aim for carbon nanoarchitectonics, as is the development of CDI and other technologies.

**Fig. 1:**
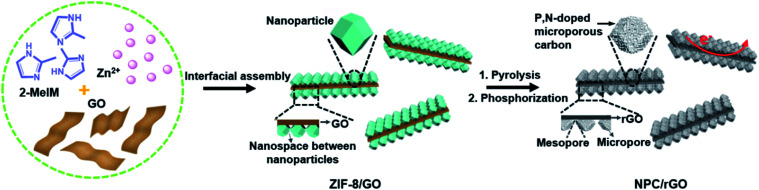
Schematic illustration for the preparation of NPC/rGO.

Herein, the interfacial assembly of metal–organic-framework (MOF) nanoparticles on 2D graphene oxide (GO) nanosheets has been developed to produce precursors for graphene–carbon 2D heterostructures having hierarchical meso–micropores and predesignated functionalities. Typically (Fig. 1), N- and Zn-rich ZIF-8 is uniformly anchored on both sides of GO nanosheets, producing 2D ZIF-8/GO nanoarchitectures, where the GO can be ultimately transformed into graphene nanosheets providing the requisite good electrical conductivity. After simple pyrolysis, ZIF-8/GO is converted to an N-doped carbon–graphene 2D heterostructured material, abbreviated NC/rGO, and the spaces between the nanoparticles anchored on the GO nanosheets spontaneously form mesopores due to the collapse of the crystals during heating. Concurrently, the evaporation of metallic Zn leads to the formation of a highly microporous structure within the carbonized nanoparticles. Finally, the subsequent phosphorization of NC/rGO with phytic acid simultaneously introduces the secondary P dopants and micropores, forming the hierarchically porous N,P-doped 2D carbon–graphene heterostructure (denoted as NPC/rGO). Pnictogen elements P and N possess the same number of valence electrons, while P atoms can also exist in hypervalent states. In addition, P atoms are much larger in diameter than C, resulting in local structural distortions of the hexagonal carbon framework with P atoms protruding out of the graphene plane, thus overcoming steric hindrance effects encountered in N-doped carbon materials.^34,38^ Consequently, the resulting NPC/rGO material exhibits several advantageous properties, including 2D architectures, P,N dual dopants, hierarchical mesopore–micropores, and good electrical conductivity, leading to an ultrahigh SAC of 39.34 mg g^−1^ with good cycling stability, significantly surpassing the previously reported benchmark performance.

## Results and discussion

**Fig. 2:**
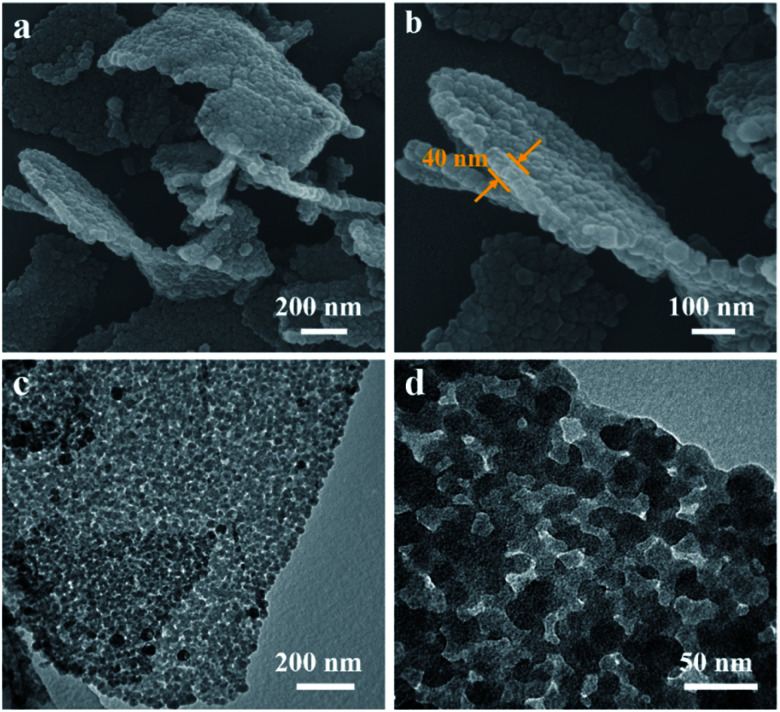
(a and b) SEM and (c and d) TEM images of ZIF-8/GO.

The structure and morphology of ZIF-8/GO was observed by using scanning electron microscopy (SEM) and transmission electron microscopy (TEM). The ZIF-8/GO precursor exhibits a planar nanosheet structure with a thickness of 30–50 nm (Fig. 2a and b). The ZIF-8 nanoparticles are tightly packed forming an ultrathin ZIF layer fully covering the GO nanosheets, thus successfully generating a ZIF-8/GO heterostructure. TEM images (Fig. 2c and d) clearly show that the ZIF-8 nanocrystals, with average diameter of about 25 nm, are coated uniformly on the GO sheets, and there are obvious nanospaces between the ZIF-8 nanoparticles. These nanospaces will be converted to mesopores in the carbonized samples. The corresponding phase of the ZIF-8/GO was identified by using X-ray diffraction (XRD, Fig. S1†); characteristic diffraction peaks could be indexed to those of pure ZIF-8 crystals indicating successful formation of ZIF-8 on GO sheets.

**Fig. 3:**
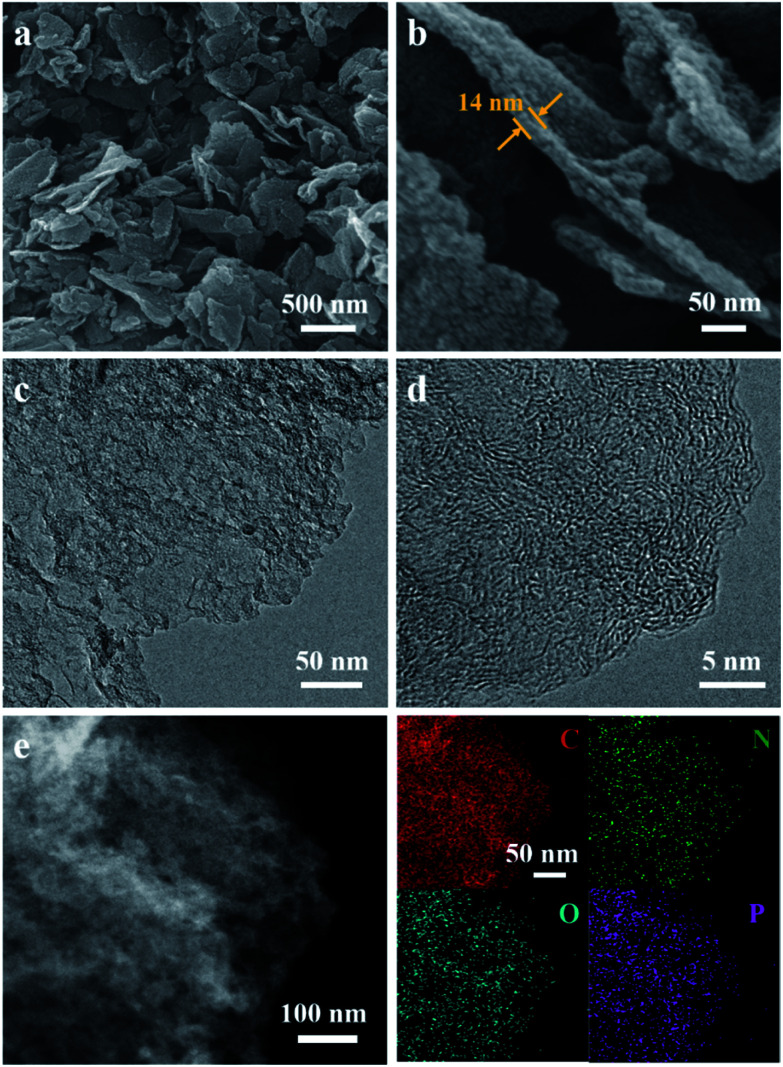
(a and b) SEM, (c) TEM, (d) HRTEM and (e) EDS elemental mapping images of NPC/rGO.

The NC/rGO hybrid was then obtained by annealing the ZIF-8@GO precursors in N_2_ atmosphere at 950 °C, leading to an ultrathin carbon nanosheet structure (Fig. S2†). After that, phytic acid was used as both the activator and phosphorus doping agent to further modify the NC/rGO at 1000 °C, with the structure and morphology of the obtained NPC/rGO remaining intact (Fig. 3a and b), indicating that the simultaneous phosphorus doping/activation procedure does not significantly affect the morphology of NC/rGO. Moreover, the coating ZIF-8 layer is transformed to a porous ultrathin carbon layer on the graphene sheet, whose thickness (about 14 nm) is about 1/3–1/2 of the original thickness of the coating layer, indicating the apparent shrinkage of the ZIF-8 nanoparticles during the stepwise heating process. It should be noted that the ZIF-8 nanoparticles also shrink in the direction parallel to the graphene surface, resulting in a large number of mesopores. A magnified TEM image (Fig. 3c) reveals that the ZIF-8-derived porous thin layer has been uniformly coated on the surface of rGO nanosheet. High-resolution TEM (HRTEM) in Fig. 3d reveals the coexistence of amorphous carbon and crystalline carbon. Energy dispersive spectroscopy (EDS) elemental mapping images (Fig. 3e) indicate uniform distributions of P and N doping heteroatoms in the NPC/rGO matrix.

**Fig. 4:**
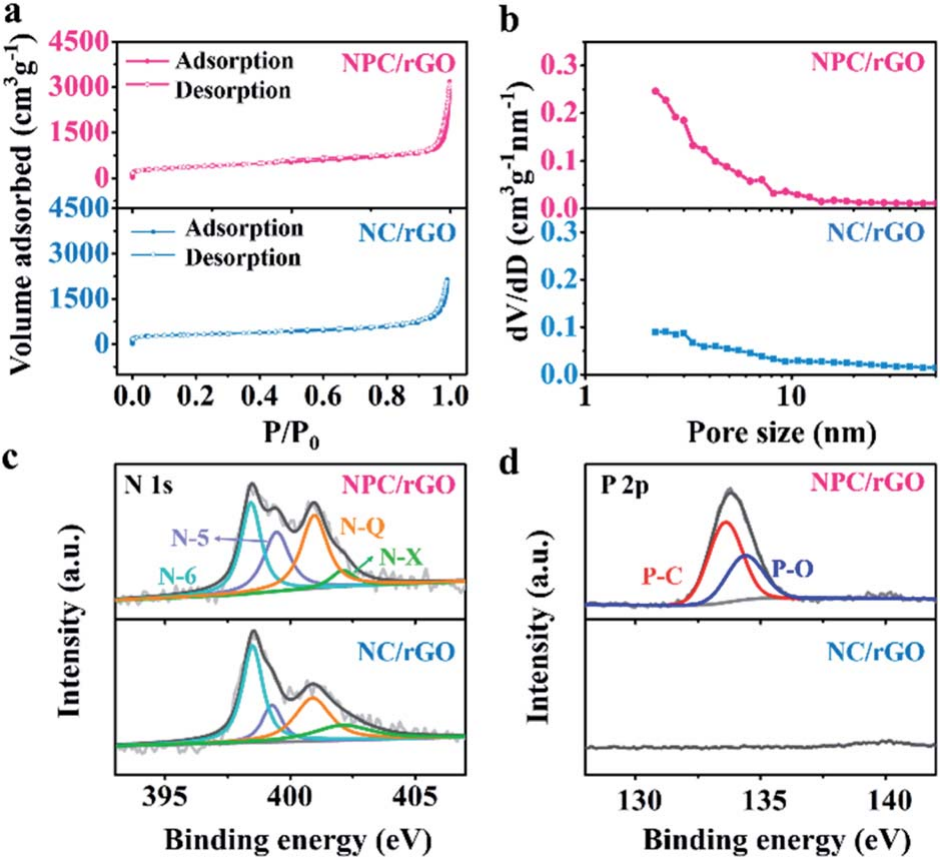
(a) N_2_ adsorption–desorption isotherms, (b) pore size distribution profiles, and high-resolution XPS spectra of (c) N 1s and (d) P 2p for NC/rGO and NPC/rGO.

The SSAs and pore structure distributions of NC/rGO and NPC/rGO were analyzed according to their N_2_ adsorption/desorption isotherms and the values compared with N-doped carbon derived from ZIF-8 nanoparticles (denoted as NC; Fig. S3†). As shown in the N_2_ adsorption/desorption plots for NC/rGO and NPC/rGO (Fig. 4a), a sharp increase in adsorption volume at a relatively low pressure (*P*/*P*_0_ < 0.1) indicates the presence of micropores within the carbon matrix. Hysteresis loops and gradual increase of adsorbed N_2_ amount at higher relative pressure imply the presence of mesopores. The hierarchical micro–mesopore structures are also clearly indicated by pore size distributions shown in Fig. 4b and are generated by the following process: (1) micropores originate not only from the pyrolysis of ZIF-8 nanoparticles where metallic Zn evaporates at high temperature leading to micropores in the carbon matrix,^39^ but also from the phosphorization process with phytic acid which also activates the carbon matrix generating further micropores.^34^ (2) Mesopores form by conversion of nanospaces between the interconnected ZIF-8 nanoparticles on NC/rGO or NPC/rGO. The collapse of the crystal frameworks during pyrolysis expands the nanospaces between particles, thus generating mesopores within the carbon matrix. Detailed information for SSA, and pore volume have been calculated and are listed in Table S1.† NPC/rGO has a larger SSA (1336 m^2^ g^−1^) than NC/rGO (1019 m^2^ g^−1^), due to the activation by phytic acid. Moreover, NPC/rGO and NC/rGO both have larger SSAs than NC (889 m^2^ g^−1^; Fig. S3†), highlighting the superiority in design of these structures.

Chemical compositions of NC/rGO and NPC/rGO were further analysed using X-ray photoelectron spectroscopy (XPS). As shown in Fig. 4c, the high-resolution N 1s spectra for NC/rGO and NPC/rGO were deconvoluted to four peaks at 398.5, 400.0, 401.0, and 402.1 eV, respectively characteristic of pyridinic-N (N-6), pyrrolic-N (N-5), graphitic-N (N-Q), and oxidized-N (N-X).^28^ Pyridinic- and pyrrolic-N can increase wettability and ion accommodation of carbon matrices, while graphitic-N improves the electrical conductivity of the carbon frameworks. Two peaks found in the high-resolution P 2p spectra (Fig. 4d) at 133.4 and 134.4 eV, respectively correspond to P–C and P–O bonds,^25^ indicating that phosphorus has been successfully doped into the carbon matrix. The corresponding P content for NPC/rGO was determined to be 3.18 at%.

**Fig. 5:**
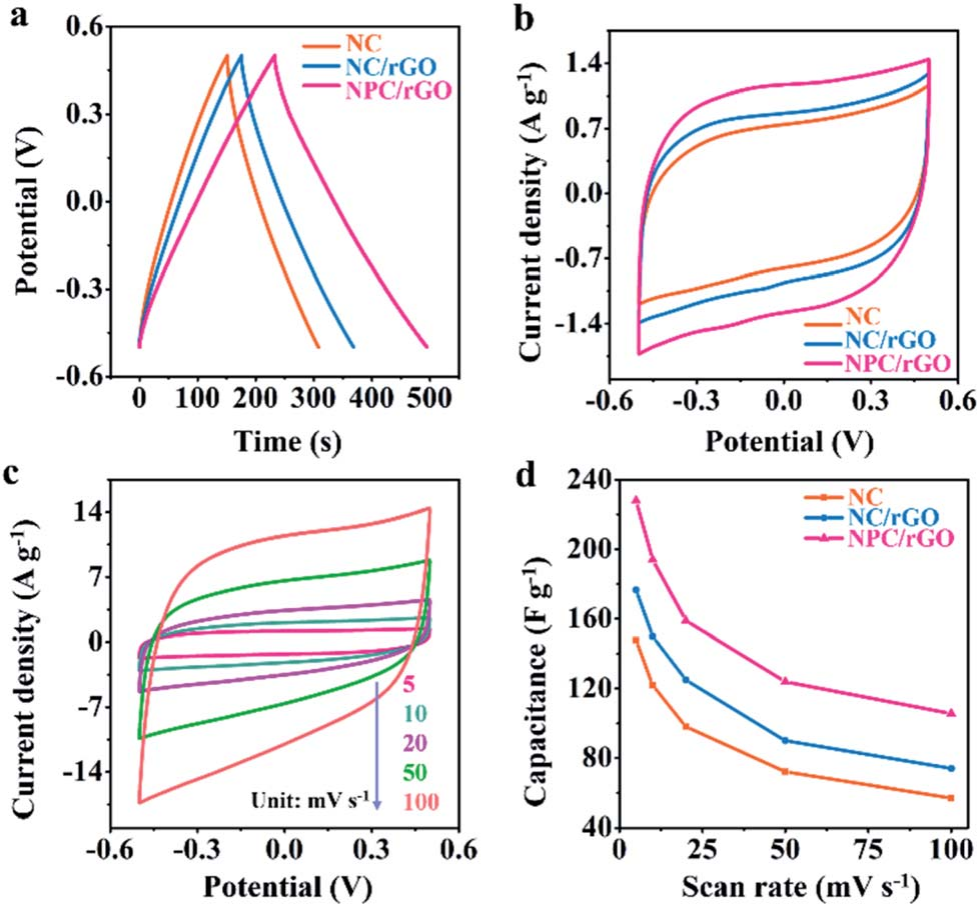
(a) GCD curves at 1 A g^−1^ and (b) CV curves at 5 mV s^−1^ of NC, NC/rGO and NPC/rGO in 1 M NaCl aqueous electrolyte. (c) CV curves of NPC/rGO at different scan rates in 1 M NaCl aqueous electrolyte. (d) Specific capacitances *vs.* scan rates of NC, NC/rGO and NPC/rGO in 1 M NaCl aqueous electrolyte.

The electrochemical properties of electrode materials were explored by cyclic voltammetric (CV) and galvanostatic charge/discharge (GCD) analyses. Fig. 5a shows the GCD curves for NC, NC/rGO and NPC/rGO electrodes in the voltage range −0.5 V to 0.5 V at 1 A g^−1^. The nearly symmetric triangular curves are slightly distorted possibly due to pseudocapacitive effects caused by the doping heteroatoms. Furthermore, NPC/rGO exhibits a longer discharge time than NC and NC/rGO, indicating its improved capacitive performance. The CV curves obtained at a scan rate of 5 mV s^−1^ for NPC/rGO, NC/rGO and NC are shown in Fig. 5b with the slightly distorted rectangular patterns, suggesting pseudocapacitive behaviour. Furthermore, the scan rate dependency of CV curves of NPC/rGO shown in Fig. 5c indicates good capacitive reversibility based on similarity in the CV curve shape at different scan rates. Fig. 5d shows the dependency of capacitance on scan rate for NC, NC/rGO and NPC/rGO. High capacitances at low scan rates are based on there being sufficient time for ions to diffuse deep into the micropores' interiors for EDL formation, along with interaction at heteroatom dopants for increased pseudocapacitance.^40,41^ In contrast, at higher scan rates, this tendency is necessarily decreased. Furthermore, the highest specific capacitance of 228 F g^−1^ is observed for NPC/rGO at 5 mV s^−1^, and the superior capacitance of NPC/rGO over the other materials persists at all the scan rates studied. This is attributed to its 2D layered heterostructure with largest SSA of the materials, its hierarchical meso–micropore structure, as well as N and P co-doping, which favours a high deionization capacity.

**Fig. 6:**
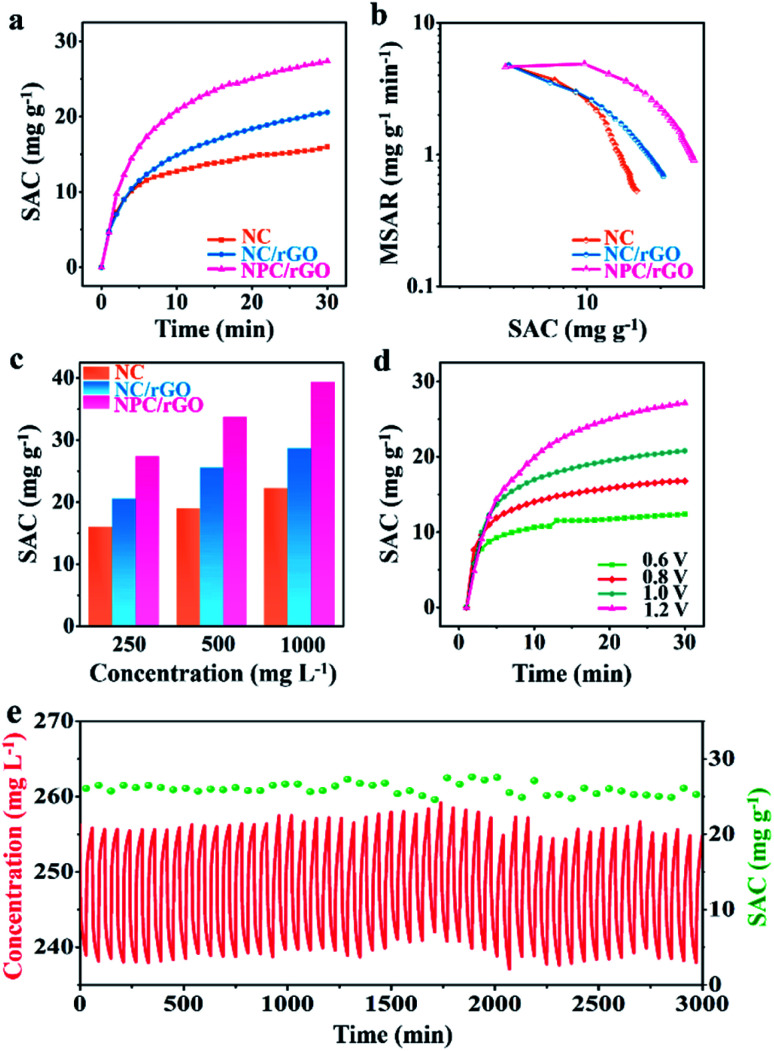
(a) Variations in SAC for the materials, (b) CDI Ragone plots and (c) SAC values at different NaCl concentrations for NC, NC/rGO and NPC/rGO. (d) SAC variations of NPC/rGO at different operating voltages. (e) Cycling desalination performance of NPC/rGO over 50 cycles.

The CDI performances of NC, NC/rGO, NPC/rGO were compared in NaCl solution with an initial concentration of 250 mg L^−1^ at 1.2 V (Fig. 6a). These data yield an SAC value of 27.36 mg g^−1^ for NPC/rGO, which is higher than those of NC/rGO (20.53 mg g^−1^) and NC (15.93 mg g^−1^), demonstrating its superior electrosorption performance. The corresponding CDI Ragone plots, *i.e.*, mean salt adsorption rate (MSAR) *vs.* SAC, are shown in Fig. 6b. These clearly indicate that NPC/rGO more effectively performs desalination at a greater rate than either NC/rGO or NC. The superior CDI performance of NPC/rGO is most likely due to its more highly appropriate pore size distribution and the presence of dual heteroatom dopants in its carbon matrix. In the designed pore architecture of NPC/rGO, the highly microporous structure provides multiple adsorption sites for the accommodation of ions,^42^ while its mesopores facilitate ion transport by establishing pathways for accelerated ion diffusion.^17^

Furthermore, the zeta potentials of NC, NC/rGO and NPC/rGO samples measured in NaCl solution at various pH values were determined to characterise surface charge properties. Typically, the pH values of seawater and drinking water are between 6 and 8.5. As illustrated in Fig. S4,† NPC/rGO has a greater negative surface charge in this pH range than either NC or NC/rGO, possibly caused by the secondary dopant P. This feature favours improved ionic adsorption. Contact angle tests were also performed to assess the hydrophilicity of carbon before and after P doping (Fig. S5†). NPC/rGO forms a much smaller contact angle with water clearly, indicating its greater hydrophilicity over NC/rGO due to the formation of hydrophilic functional groups such as P–O in the P-doped carbon matrix.^28^ As a consequence of its superior structural features including 2D nanoarchitectures with well-designed meso–micropores, more highly negatively-charged surfaces, and excellent hydrophilicity, NPC/rGO displays the best CDI properties of the three materials studied.

CDI performances of the three electrodes were further investigated at different NaCl solution concentrations, and the corresponding SAC results are shown in Fig. 6c. It can be seen that with the increase of NaCl concentration, SAC increases for all samples, and a maximum SAC of 39.34 mg g^−1^ is achieved for NPC/rGO in 1000 mg L^−1^ NaCl solution at 1.2 V. To the best of our knowledge, this value represents the state-of-the-art performance reported for porous carbon desalinator materials (Table S2†). Fig. 6d shows SACs of NPC/GO at different operating voltages from 0.6 to 1.2 V. Increasing the operating voltage leads to improved CDI performance. NPC/rGO maintains a high SAC of 12.5 mg g^−1^ even at a very low operating voltage of 0.6 V. Cycling CDI test over 50 cycles was also conducted to evaluate the cycling stability of the NPC/rGO electrode. As shown in Fig. 6e, the NPC/rGO electrode exhibits excellent cycling stability without obvious degradation of performance. Furthermore, the chemical composition of NPC/rGO after 50 cycles tests was investigated by XPS (Fig. S6 and S7†). Content and type of N 1s (Fig. S6a†) and P 2p (Fig. S6b†) species had not changed significantly after 50 cycles. On the other hand, for C 1s spectra (Fig. S7†), the relative peak intensity of C–O and C=O species slightly increases, accompanied by a decrease in carbon content from 79.39 at% to 73.06 at%. Despite this partial electrode oxidation during the CDI cycling process, the overall composition of the NPC/rGO electrode varied only slightly, revealing the stability and practicability of our materials.

## Conclusions

In summary, we have successfully fabricated an N,P-doped carbon–graphene heterostructure having a hierarchically meso-/microporous layered architecture by the phosphorization of carbonized ZIF-8/GO nanosheets using phytic acid. The resulting NPC/rGO has a 2D layered nanosheet structure with large SSA, appropriate pore structure, enhanced electrical conductivity and abundant N,P heteroatom dopants. As a result, NPC/rGO achieves an excellent CDI performance with a high SAC of 39.34 mg g^−1^ and good cycling stability. To the best of our knowledge, this value is also larger than those of most of the carbon electrodes reported to date. This work not only establishes a promising CDI electrode material for desalination applications, but also showcases the significance of nanoparticle-driven assembly nanoarchitectonics in the design of graphene–carbon 2D heterostructures.

## Data availability

The data that support the findings of this study are available within the article and its ESI, or from the corresponding authors on reasonable request.

## Author contributions

J. G. conducted most of the experiments, collected/analyzed the data, and wrote the initial manuscript. X. X. conceived the idea, supervised the project, and wrote/edited the manuscript. L. W., J. D., Y. K., Y. L. assisted during data analysis and some of the experiments. J. P. H. reviewed and edited the manuscript. W. G. and Y. Y. supervised the project and edited the manuscript. All authors reviewed and commented on the manuscript.

## Conflicts of interest

There are no conflicts to declare.

## Supplementary Material

SC-012-D1SC00915J-s001
