# Supplementary material for: Graphene–carbon 2D heterostructures with hierarchically-porous P,N-doped layered architecture for capacitive deionization
Source: Chem Sci. 2021 Jun 30;12(30):10334–40. doi: 10.1039/d1sc00915j (PMC8336432; doi:10.1039/d1sc00915j)
Supplement: SC-012-D1SC00915J-s001 [file SC-012-D1SC00915J-s001.pdf]

Electronic Supplementary Material (ESI) for Chemical Science.
This journal is © The Royal Society of Chemistry 2021

Experimental section

Materials

Graphite powder, $KMnO_4$, H_2SO_4 and HNO_3, poly(vinylidene fluoride) (PVDF), N-methyl-2-pyrrolidinone (NMP) were purchased from Wako Pure Chemical Industry. $Zn(NO_3)_2{\cdot}6H_2O$, 2-methylimidazole (2-MeIM), and phytic acid (PA) solution 50 % (*w/w*) in H_2O were purchased from Sigma-Aldrich. All chemicals were used as received without further purification.

Preparation of graphene oxide (GO)

In brief, graphite powder was added to a solution of concentrated HNO_3 and H_2SO_4 (1:2 *v/v*) and maintained at 80 °C for 5 h. The mixture was then allowed to cool to room temperature, deionized (DI) water was added, and the mixture allowed to stand overnight. The reaction vessel was then placed in an ice bath, $KMnO_4$ was slowly added. Subsequently, the mixture was allowed to stand with stirring for 2 h. After dilution with DI water, 30% H_2O_2 was added to the mixture, and the colour of the mixture changed to brilliant yellow, accompanied by bubbling. The mixture was then filtered and the solid washed with aqueous HCl solution (1:10 *v/v*), DI, and ethanol. Finally, GO was obtained by freeze drying.

Preparation of ZIF-8/GO

Briefly, $Zn(NO_3)_2{\cdot}6H_2O$ (0.366 g) and 2-MeIM (0.811 g) were dissolved in methanol (12 mL and 20 mL, resp.). The methanolic $Zn(NO_3)_2$ solution (12 mL) was then added to the 2-MeIM solution to obtain a clear solution. GO solution (1 mg mL^{-1} in water, 8 mL) was immediately added to the $Zn(NO_3)_2$/2-MeIM mixed solution. After stirring for 20 min, the gray precipitate was collected by centrifugation (8000 rpm for 5 min) and was washed three times with methanol. Zeolitic imidazolate framework-8 ZIF-8/GO was obtained after freeze-drying.

Preparation of NC/rGO

ZIF-8/GO nanosheets were converted to NC/rGO nanosheets by carbonization at 950 °C for 2 h under a N_2 atmosphere. The ramping rate was 1 °C min^{-1} in the first stage (below 350 °C) and increased to 5 °C min^{-1} in the second stage (350 to 950 °C). For comparison, ZIF-8 was obtained without adding the GO solution and employed as the precursor to NC by direct carbonization with the same process.

Preparation of porous NPC/rGO

NPC/rGO nanosheets were prepared by post-activation with PA. In a typical activation process, NC/rGO (30.0 mg) were dispersed in ethanol (1.5 mL) containing PA solution (170 μL). The suspension was then transferred to a quartz boat and dried at 60 °C. Subsequently, the quartz boat was placed in a quartz tube and heated under a N_2 atmosphere. The temperature was increased to 350 °C then 550 ℃ at a ramp rate of 5 °C min^{-1}. Each stage was held for 0.5 h. Temperature was then increased to 1000 °C and held for another 2 h.

Characterization.

Crystal identity of the samples was characterized by using powder X-ray diffraction (XRD, Ultima Rint 2000 X-ray diffractometer, RIGAKU, Japan) measurements using Cu Kα radiation (40 kV, 40 mA, 2° min^{-1} scan rate). Morphologies of the samples were observed by field-emission scanning electron microscopy (FESEM, HITACHI SU8000). Mesoporous structure of the samples was confirmed by transmission electron microscopy (TEM, JEOL JEM-2100). N_2 adsorption-desorption isotherms were obtained by using a Belsorp-max (BEL,Japan) instrument where the specific surface areas and pore size distributions were analyzed by the Brunauer-Emmett-Teller (BET) method and Barrett-Joyner-Halenda (BJH) models, respectively. X-ray photoelectron spectroscopy (XPS, Axis Ultra, Kratos Analytical Ltd) was used to investigate the chemical state of phosphorus and nitrogen in the samples. The Zeta potential measurements were done in aqueous solutions of the samples in function of pH values ranging from 3 to 11 by Zeta sizer Nano-ZS90 (Malvern,

United Kingdom).

Electrochemical analyses

The electrochemical measurements were performed on CHI 660E electrochemical workstation in a three-electrode system with 1 M NaCl aqueous electrolyte. A platinum (Pt) wire and Hg/HgO were selected as the counter electrode and reference electrode, respectively. Cyclic voltammetry (CV) and gravimetric charge-discharge (GCD) measurements were carried out in the potential range of -0.5 to 0.5 V.

The specific capacitances (C, F g^{-1}) were calculated from the CV curves by using the following equation:

$$C = \int idV \big/ 2\Delta V m v \tag{1}$$

where i is the discharge current density (A), m is the mass of active materials (g), , and ΔV is the voltage window (V), v is the scan rate (V s^{-1}).

Desalination analysis

Each individual CDI electrode was fabricated by depositing a mixture of the sample, Super-P and PVDF binder with a weight ratio of 8:1:1. The mixture was pressed onto graphite paper (2.5 × 2.5 cm^2) and dried under reduced pressure at 60 °C for 12 h.

CDI tests were conducted using a batch-mode with continuous recycling system, which consists of a pair of anion- and cation-exchange membranes, a peristaltic pump, a power source, and a tank. The real-time saline concentration variation was monitored and measured by using an ion conductivity meter. The volume of the saline solution was fixed at 30 mL, the flow rate was 30 mL min^{-1}, and the operating voltage was 1.2 V.

The salt adsorption capacity (Γ, mg g^{-1}) and mean salt adsorption rate ($MSAR$, mg g^{-1} min^{-1}) at t min were calculated as follows:

$$\Gamma = (C_0 - C_t) \times V/m \tag{2}$$

$$MSAR = \Gamma/t \tag{3}$$

where C_0 and C_t represent the concentrations of NaCl at the initial stage and t min, respectively (mg L^{-1}); V is the volume of the NaCl solution (L); and m represents the total mass of the electrode materials (g).

Supplementary Figures and Tables

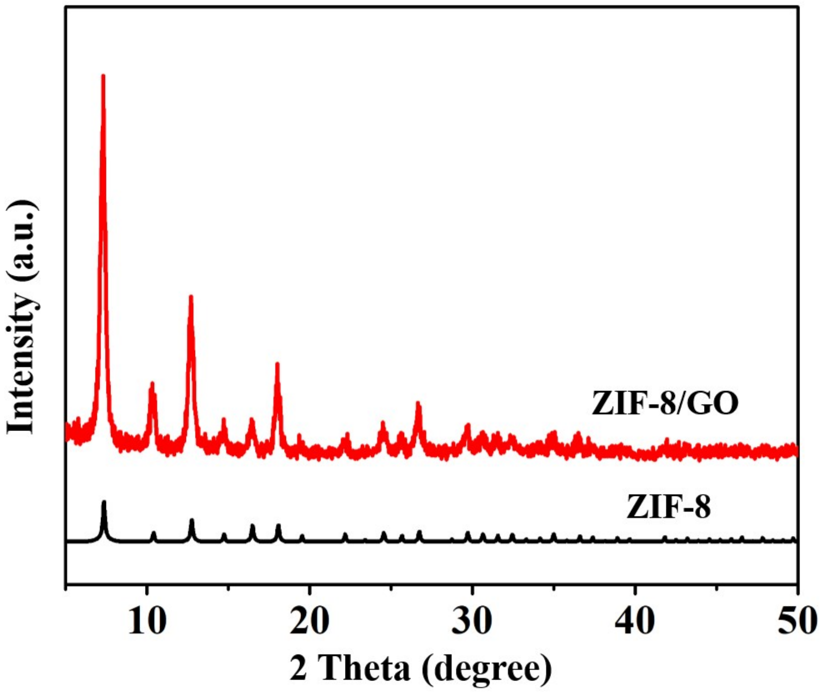

Fig. S1 XRD patterns of ZIF-8 and ZIF-8/GO.

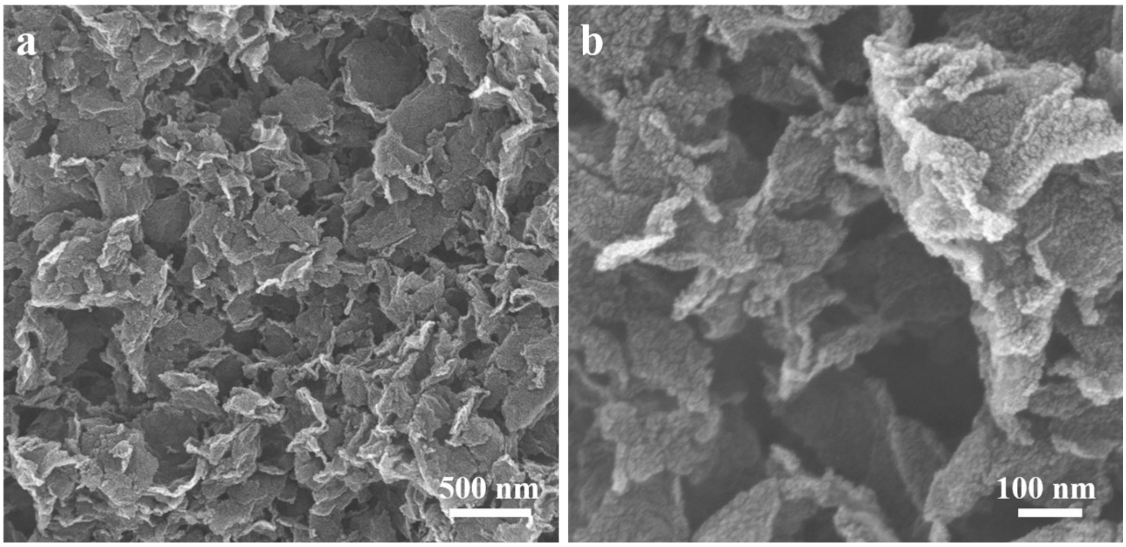

Fig. S2 (a) Low-resolution and (b) high-resolution SEM images of NC/rGO.

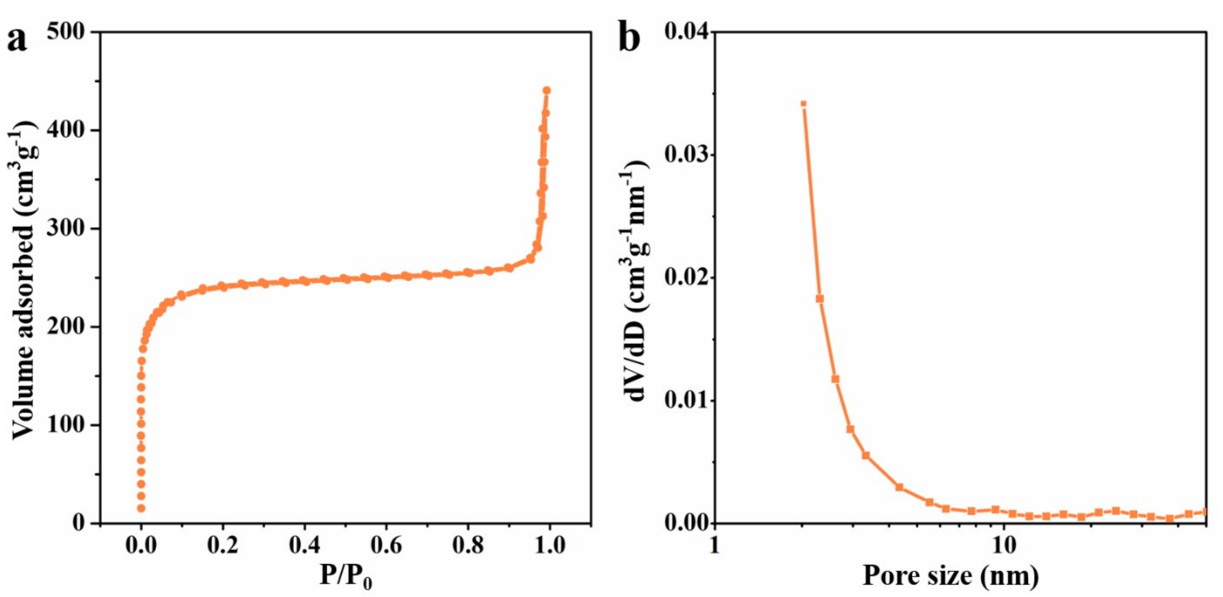

Fig. S3 (a) N_2 adsorption-desorption isotherms and (b) pore size distribution profiles of NC.

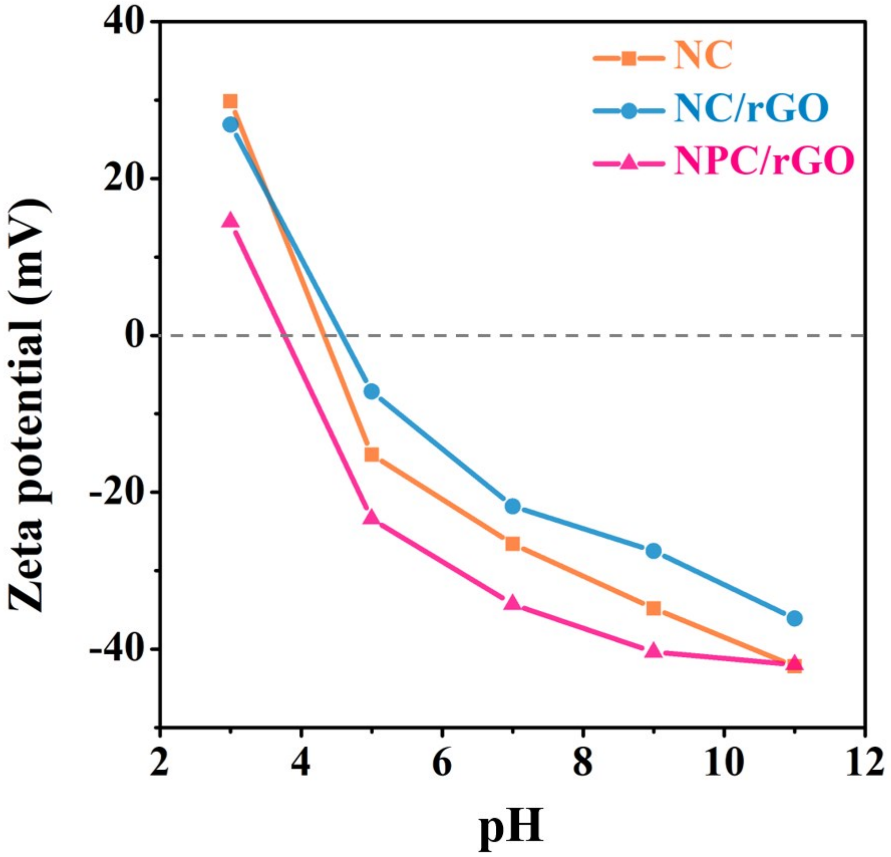

Fig. S4 Zeta potential curves of NC, NC/rGO and NPC/rGO in NaCl solution under varying pH.

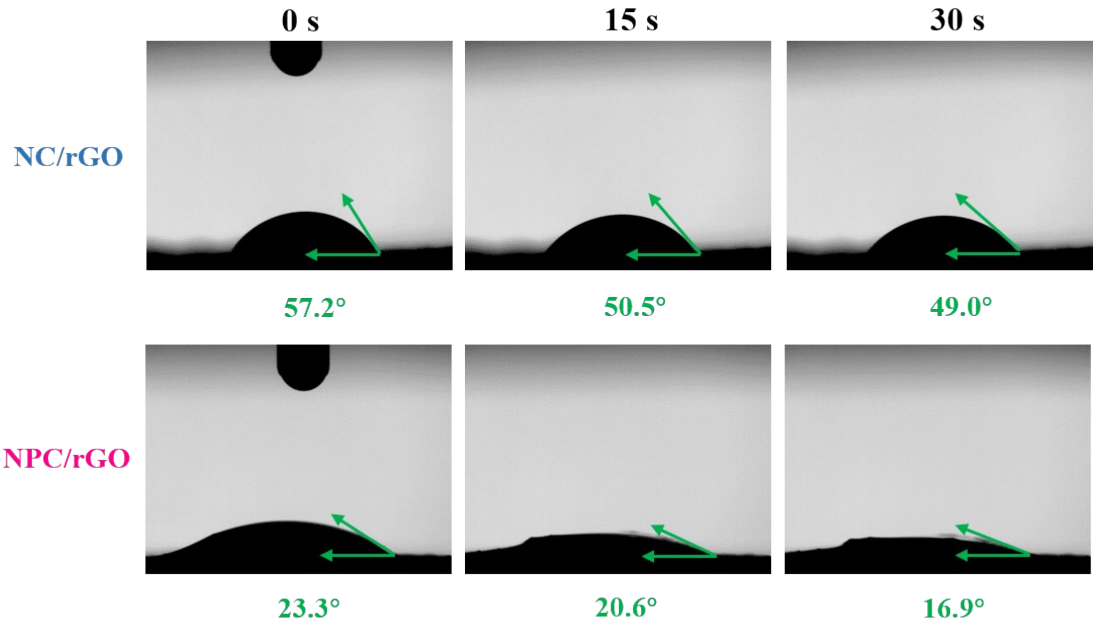

Fig. S5 Water contact angles of NC/rGO and NPC/rGO electrodes.

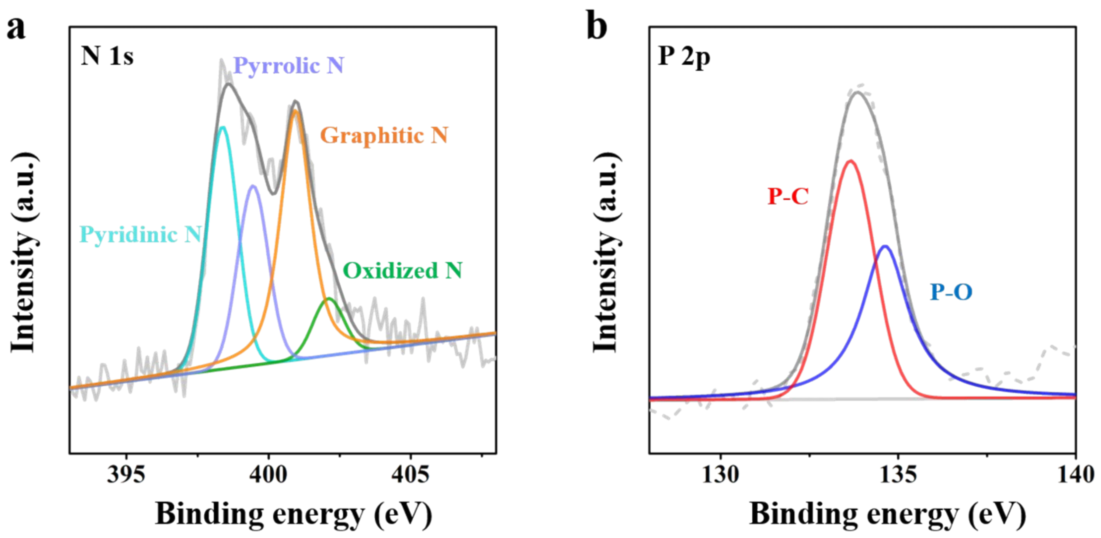

Fig. S6 (a) N 1*s* and (b) P 2*p* spectra of NPC/rGO after 50 cycles.

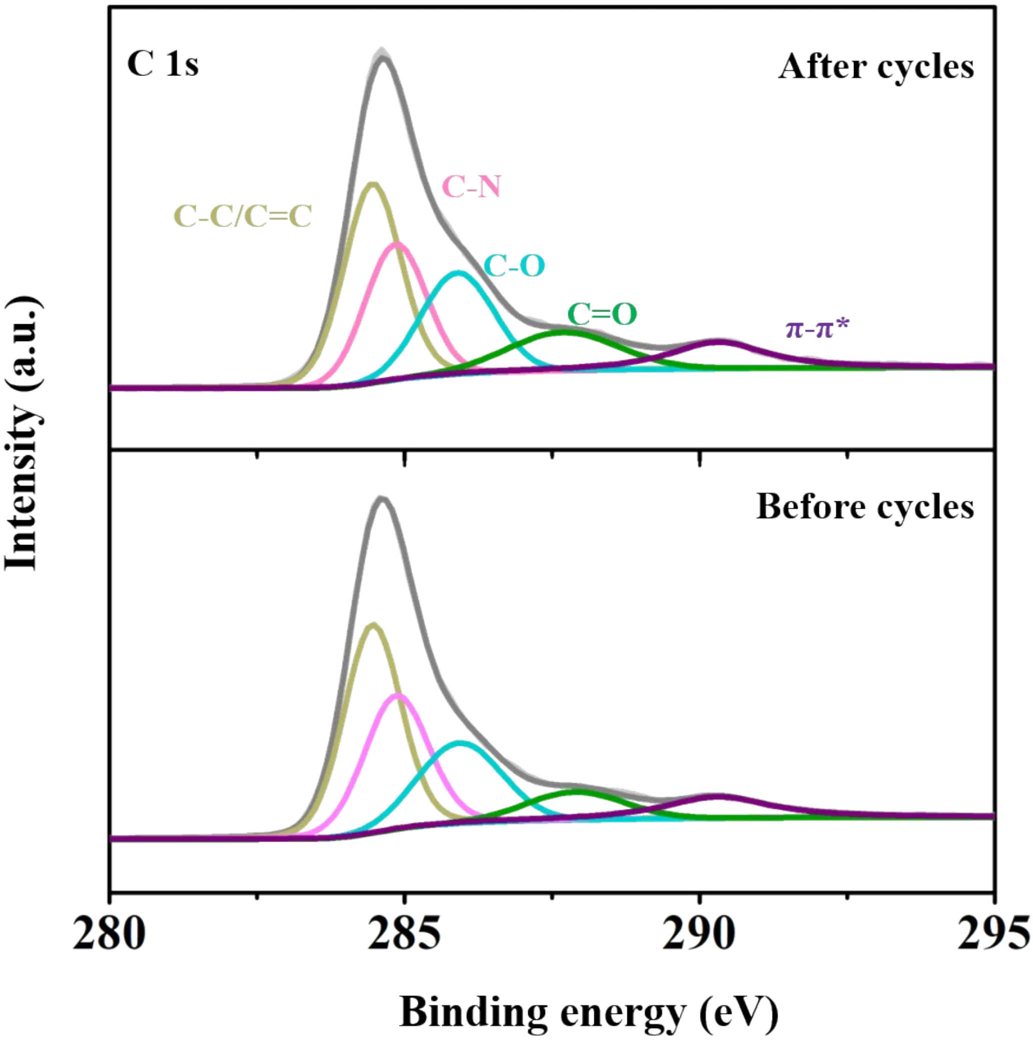

Fig. S7 C 1*s* spectra of NPC/rGO before and after 50 cycles.

Table S1. SSAs and pore volumes of NC, NC/rGO and NPC/rGO

Sample	SSA (m^2 g^{-1})	Pore volume (cm^3 g^{-1})
NC	889	0.42
NC/rGO	1019	3.29
NPC/rGO	1336	3.09

Table S2. Performance comparisons of NPC/rGO and other carbon materials

Sample	SSA ($m^2 g^{-1}$)	Voltage (V)	SAC ($mg g^{-1}$)	Ref.
P-60	1260	1.5	5.28	S1
Filtrasorb 400	964	1.0	13.03	S2
AC-1-2.0	2105	1.0	9.72	S3
C5A85K4	3649	1.2	22.20	S4
HPC	609	1.2	10.27	S5
CCS	2680	1.2	16.10	S6
3DHCA	2061	1.2	17.83	S7
PCNSs	2853	1.1	15.60	S8
NPC	1036.2	1.2	15.50	S9
SBB-CO2-30	1019	1.2	28.90	S10
CTS-AC	2727	1.2	14.12	S11
PCS1000	1321	1.6	5.81	S12
NPCSs1000	1640	1.2	14.91	S13
N-PHCS	512	1.4	12.95	S14
hCSs-800	1529	1.2	15.80	S15
PCSs-800	485.6	1.2	18.50	S16
CHS-1	809.91	1.6	18.88	S17
N-HMCSs	1099	1.6	16.60	S18
OMC	844	1.2	0.68	S19
NMCs	842.3	1.2	20.63	S20
NOMC	459.32	1.6	26.20	S21
OMC-O	1481	1.2	9.80	S22
o-OMCs-1000	780.3	1.2	14.58	S23
OMC-S	1491	1.2	0.93	S24
ACk2	1968	1.6	11.70	S25
N-HMCS/HGH	337.7	1.4	37.20	S26
GSSNA-11	664	1.2	22.09	S27

CSG	711.9	1.5	9.60	S28
GE/MC	685.2	2.0	0.73	S29
MC	1700	1.2	3.50	S30
Activated graphene	3513	2.0	11.86	S31
G@MC-O-thin	1270	1.2	24.30	S32
AGE-30	898	1.2	6.26	S33
RGO/AC	779	1.2	2.94	S34
GTAC	426.56	1.2	10.94	S35
GS	356.0	1.2	14.90	S36
mGE	474.0	1.2	14.20	S37
HGF	124	2.0	29.60	S38
EPD-CNTs	82	1.2	2.33	S39
CNTs/CNFs	211	1.2	1.61	S40
MWCNT/PVA	208	1.2	13.07	S41
nit-CNTs	200.9	1.2	17.18	S42
OMC/CNT	620.9	1.2	0.63	S43
ZFCarbon	2060	1.2	8.10	S44
e-CNF-PCP	1450.6	1.2	12.56	S45
PC-900	1563.09	1.2	9.39	S46
PC-900	1911	1.2	10.90	S47
NC-800	798	1.2	8.52	S48
PCP1200	1187.8	1.2	13.86	S49
ZIF-8@PZS-C	929	1.2	22.19	S50
aG10P	1067	1.2	36.10	S51
**NPC/rGO**	**1336**	**1.2**	**39.34**	**This work**

References

S1. Y.-J. Kim and J.-H. Choi, *Sep. Purif. Technol.*, 2010, **71**, 70-75.

S2. C.-H. Hou and C.-Y. Huang, *Desalination*, 2013, **314**, 124-129.

S3. C.-L. Yeh, H.-C. Hsi, K.-C. Li and C.-H. Hou, *Desalination*, 2015, **367**, 60-68.

S4. R. L. Zornitta, K. M. Barcelos, F. G. E. Nogueira and L. A. M. Ruotolo, *Carbon*, 2020, **156**, 346-358.

S5. Y. Li, I. Hussain, J. Qi, C. Liu, J. Li, J. Shen, X. Sun, W. Han and L. Wang, *Sep. Purif. Technol.*, 2016, **165**, 190-198.

S6. G.-X. Li, P.-X. Hou, S.-Y. Zhao, C. Liu and H.-M. Cheng, *Carbon*, 2016, **101**, 1-8.

S7. S. Zhao, T. Yan, H. Wang, J. Zhang, L. Shi and D. Zhang, *ACS Appl. Mater. Interfaces*, 2016, **8**, 18027-18035.

S8. T. Wu, G. Wang, Q. Dong, F. Zhan, X. Zhang, S. Li, H. Qiao and J. Qiu, *Environ. Sci. Technol.*, 2017, **51**, 9244-9251.

S9. C. Zhao, G. Liu, N. Sun, X. Zhang, G. Wang, Y. Zhang, H. Zhang and H. Zhao, *Chem. Eng. J.*, 2018, **334**, 1270-1280.

S10. Y.-H. Tang, S.-H. Liu and D. C. W. Tsang, *J. Hazard. Mater.*, 2020, **383**, 121192.

S11. Q. Wu, D. Liang, X. Ma, S. Lu and Y. Xiang, *RSC Adv.*, 2019, **9**, 26676-26684.

S12. Y. Liu, L. Pan, T. Chen, X. Xu, T. Lu, Z. Sun and D. H. C. Chua, *Electrochim. Acta*, 2015, **151**, 489-496.

S13. Y. Liu, T. Chen, T. Lu, Z. Sun, D. H. C. Chua and L. Pan, *Electrochim. Acta*, 2015, **158**, 403-409.

S14. S. Zhao, T. Yan, H. Wang, G. Chen, L. Huang, J. Zhang, L. Shi and D. Zhang, *Appl. Surf. Sci.*, 2016, **369**, 460-469.

S15. X. Xu, H. Tang, M. Wang, Y. Liu, Y. Li, T. Lu and L. Pan, *J. Mater. Chem. A*, 2016, **4**, 16094-16100.

S16. Y. Li, X. Xu, S. Hou, J. Ma, T. Lu, J. Wang, Y. Yao and L. Pan, *Chem. Commun.*, 2018, **54**, 14009-14012.

S17. Z. Y. Leong and H. Y. Yang, *RSC Adv.*, 2016, **6**, 53542-53549.

S18. Y. Li, J. Qi, J. Li, J. Shen, Y. Liu, X. Sun, J. Shen, W. Han and L. Wang, *ACS Sustain. Chem. Eng.*, 2017, **5**, 6635-6644.

S19. L. Zou, L. Li, H. Song and G. Morris, *Water Res.*, 2008, **42**, 2340-2348.

S20. X. Xu, A. Enaiet Allah, C. Wang, H. Tan, A. A. Farghali, M. Hamdy Khedr, V. Malgras, T. Yang and Y. Yamauchi, *Chem. Eng. J.*, 2019, **362**, 887-896.

S21. S. Tian, J. Wu, X. Zhang, K. Ostrikov and Z. Zhang, *Chem. Eng. J.*, 2020, **380**, 122514.

S22. F. Duan, X. Du, Y. Li, H. Cao and Y. Zhang, *Desalination*, 2015, **376**, 17-24.

S23. X. Xu, H. Tan, Z. Wang, C. Wang, L. Pan, Y. V. Kaneti, T. Yang and Y. Yamauchi, *Environ. Sci. Nano*, 2019, **6**, 981-989.

S24. L. Li, L. Zou, H. Song and G. Morris, *Carbon*, 2009, **47**, 775-781.

S25. G. Wang, B. Qian, Q. Dong, J. Yang, Z. Zhao and J. Qiu, *Sep. Purif. Technol.*, 2013, **103**, 216-221.

S26. M. Mi, X. Liu, W. Kong, Y. Ge, W. Dang and J. Hu, *Desalination*, 2019, **464**, 18-24.

S27. Z. U. Khan, T. Yan, L. Shi and D. Zhang, *Environ. Sci. Nano*, 2018, **5**, 980-991.

S28. L. Chang and Y. Hang Hu, *J. Colloid Interf. Sci.*, 2019, **538**, 420-425.

S29. D. Zhang, X. Wen, L. Shi, T. Yan and J. Zhang, *Nanoscale*, 2012, **4**, 5440-5446.

S30. C. Tsouris, R. Mayes, J. Kiggans, K. Sharma, S. Yiacoumi, D. DePaoli and S. Dai, *Environ. Sci. Technol.*, 2011, **45**, 10243-10249.

S31. Z. Li, B. Song, Z. Wu, Z. Lin, Y. Yao, K.-S. Moon and C. P. Wong, *Nano Energy*, 2015, **11**, 711-718.

S32. O. Noonan, Y. Liu, X. Huang and C. Yu, *J. Mater. Chem. A*, 2018, **6**, 14272-14280.

S33. Y. Zhang, L. Chen, S. Mao, Z. Sun, Y. Song and R. Zhao, *J. Colloid Interface Sci.*, 2019, **536**, 252-260.

S34. H. Li, L. Pan, C. Nie, Y. Liu and Z. Sun, *J. Mater. Chem.*, 2012, **22**, 15556-15561.

S35. G. Zhu, W. Wang, X. Li, J. Zhu, H. Wang and L. Zhang, *RSC Adv.*, 2016, **6**, 5817-5823.
S36. X. Xu, L. Pan, Y. Liu, T. Lu, Z. Sun and D. H. C. Chua, *Sci. Rep.*, 2015, **5**, 8458.
S37. X. Xu, Y. Liu, M. Wang, X. Yang, C. Zhu, T. Lu, R. Zhao and L. Pan, *Electrochim. Acta*, 2016, **188**, 406-413.
S38. J. Li, B. Ji, R. Jiang, P. Zhang, N. Chen, G. Zhang and L. Qu, *Carbon*, 2018, **129**, 95-103.
S39. C. Nie, L. Pan, H. Li, T. Chen, T. Lu and Z. Sun, *J. Electroanal. Chem.*, 2012, **666**, 85-88.
S40. H. Li, L. Pan, Y. Zhang, L. Zou, C. Sun, Y. Zhan and Z. Sun, *Chem. Phys. Lett.*, 2010, **485**, 161-166.
S41. C.-H. Hou, N.-L. Liu, H.-L. Hsu and W. Den, *Sep. Purif. Technol.*, 2014, **130**, 7-14.
S42. P. Shi, C. Wang, J. Sun, P. Lin, X. Xu and T. Yang, *Sep. Purif. Technol.*, 2020, **235**, 116196.
S43. Z. Peng, D. Zhang, L. Shi and T. Yan, *J. Mater. Chem.*, 2012, **22**, 6603-6612.
S44. M. Wang, X. Xu, Y. Liu, Y. Li, T. Lu and L. Pan, *Carbon*, 2016, **108**, 433-439.
S45. Y. Liu, J. Ma, T. Lu and L. Pan, *Sci. Rep.*, 2016, **6**, 32784.
S46. L. Chang, J. Li, X. Duan and W. Liu, *Electrochim. Acta*, 2015, **176**, 956-964.
S47. X. Duan, W. Liu and L. Chang, *J. Taiwan Inst. Chem. E.*, 2016, **62**, 132-139.
S48. N.-L. Liu, S. Dutta, R. R. Salunkhe, T. Ahamad, S. M. Alshehri, Y. Yamauchi, C.-H. Hou and K. C. W. Wu, *Sci. Rep.*, 2016, **6**, 28847.
S49. Y. Liu, X. Xu, M. Wang, T. Lu, Z. Sun and L. Pan, *Chem. Commun.*, 2015, **51**, 12020-12023.
S50. J. Zhang, J. Fang, J. Han, T. Yan, L. Shi and D. Zhang, *J. Mater. Chem. A*, 2018, **6**, 15245-15252.
S51. Z. Y. Leong, G. Lu and H. Y. Yang, *Desalination*, 2019, **451**, 172-181.
